# Optical and Photonic Materials: From Passive Media to Tunable, Engineered, and Application-Driven Platforms

**DOI:** 10.3390/ma19142955

**Published:** 2026-07-09

**Authors:** Ruxue Wei, Weili Zhang

**Affiliations:** School of Electrical and Computer Engineering, Oklahoma State University, Stillwater, OK 74078, USA; ruxue.wei@okstate.edu

## 1. Introduction and Scope

Optical and photonic materials play a central role in generating, guiding, detecting, and sensing light. In recent years, the field has moved beyond viewing materials mainly as passive optical media. Increasingly, materials are being designed as active and functional platforms, with optical responses that can be tuned, reconfigured, or engineered through composition, structure, and device architecture. This selection of Editor’s Choice articles from the “Optical and Photonic Materials” Section of *Materials* reflects this shift. The collected papers cover tunable photonic media, symmetry- and topology-controlled light fields, low-dimensional and crystalline systems, and devices for light emission, detection, and sensing. They show a clear trend: optical and photonic materials are increasingly described not only by their intrinsic properties, but also by the functions they can be designed to perform. Rather than providing an exhaustive survey, this Editorial groups the selected articles into four themes and highlights the links among them, with the aim of outlining several directions in which optical and photonic materials research is moving.

## 2. An Overview of the Published Articles

### 2.1. Tunable, Reconfigurable, and Engineered Light-Field Control

Dynamic control of optical response is a key direction in modern photonics, especially for devices whose function must be adjusted after fabrication. A recent review of thermo-optic and electro-optic materials [1] summarizes how refractive-index modulation has supported tunable photonic systems, with thermo-optic materials offering broad compatibility and electro-optic materials supporting faster modulation. In parallel, the device requirements for optical interconnects to silicon chips have motivated continued development of compact and efficient modulators [2], while integrated lithium-niobate platforms have demonstrated high-speed electro-optic modulation at CMOS-compatible voltages [3]. Liquid-crystal-based tuning remains an important route in this area. For example, multi-fluorinated, high-refractive-index liquid crystals have been used to improve the electro-optical performance of polymer-dispersed liquid-crystal cells [Contribution 1]. This result is consistent with a broader principle in liquid-crystal physics: molecular structure and orientational order can strongly influence macroscopic optical response [4]. Similar tuning concepts are also being extended to the terahertz regime. A liquid-crystal-tuned topological photonic crystal, for instance, demonstrates dual-broadband edge states whose spectral response and propagation behavior can be tuned by an external field [5]. This result connects material tunability with topological light control [6] and points to a practical route for reconfigurable terahertz devices. Reconfigurability can also be achieved through structural and material engineering beyond purely electrical tuning. Near-field studies on terahertz metasurfaces [7] show that dynamically tunable architecture provide routes toward reconfigurable terahertz responses. They also highlight the importance of near-field characterization for confirming how these reconfigurable responses actually evolve. These studies show that tunable photonic platforms increasingly rely on the combined design of material response, device geometry, and field distribution.

### 2.2. Symmetry, Chirality, and Non-Hermitian Control

Beyond tuning the strength of an optical response, photonic materials can also be designed to control its form, including directionality, polarization, and modal topology. Symmetry, chirality, and non-Hermitian physics have therefore become useful design principles rather than passive material constraints. Directional coupling of surface plasmon polaritons at exceptional points [8], for example, shows that operating a plasmonic system at a non-Hermitian exceptional point can produce strongly asymmetric surface-wave launching in the visible range. This result connects to broader developments in non-Hermitian photonics and exceptional-point physics [9,10,11], where gain, loss, and coupling strength enable optical responses that are difficult to realize in purely Hermitian systems. Chirality provides another route for symmetry-based optical control. In hybrid perovskite single crystals [12], the incorporation of chiral organic ligands can break inversion symmetry and transfer chirality to the inorganic perovskite framework. This structural chirality gives rise to chiroptical responses, including circularly polarized light detection, photoluminescence polarization, and nonlinear optical responses [12,13]. These examples show that symmetry engineering can shape not only the magnitude of light–matter interaction, but also the direction, polarization, and modal structure of optical fields.

### 2.3. Structural Engineering of Low-Dimensional, Perovskite, and Crystalline Materials

Many of the functions discussed above ultimately depend on how materials are synthesized, assembled, and characterized at small length scales. A review of two-dimensional group IV monochalcogenides [14] surveys the synthesis, properties, and applications of this emerging material family, whose low symmetry and in-plane anisotropic electronic and optical properties make them attractive for anisotropic optoelectronic applications. This development builds on the broader progress of two-dimensional materials, from graphene [15] to transition metal dichalcogenides [16]. Halide-assisted synthesis of V-WSe_2_ [Contribution 2] further shows how chemical growth strategies can control vanadium incorporation into a two-dimensional host and expand its compositional design space. Perovskites provide another structurally versatile platform. Ligand-mediated, temperature-tuned growth of CsPbBr_3_ nanosheets enables ordered superlattice assembly [17], echoing the bright, composition-tunable emission demonstrated in CsPbX_3_ nanocrystals [18] and the defect tolerance that underpins their optoelectronic promise [19]. Structure–property relationships are also central to organic semiconductors, where ordered nanostructures assembled from semiconducting polymers link molecular order to carrier mobility, electrical conductivity, and photovoltaic response [Contribution 3].

### 2.4. Emission, Detection, Sensing, and Device Integration

The value of engineered materials is ultimately measured in devices. On the emission side, monolithic GaN-based dual-quantum-well light-emitting diodes achieve size-controlled, color-tunable white-light emission from a single chip [20], extending the development of efficient nitride lighting [21] toward integrated, color-tunable sources. Detection and sensing run in parallel: pyro-phototronic photodetectors [Contribution 4] combine pyroelectric and photoelectric effects to enhance photodetector performance, as part of a wider effort to engineer nanostructured materials for photon detection [22]. Scintillation-crystal detectors further enable in situ dose measurements during brachytherapy procedures [23], showing how fiber-optic crystal dosimeters can serve medical applications and building on broader advances in scintillator materials for radiation detection [24]. Device integration increasingly requires materials that are compatible with flexible, biocompatible, or mechanically compliant platforms. Spin-coated silk fibroin thin films for bioelectronic capacitors [Contribution 5] align with the broader development of flexible and stretchable electronics for biointegrated devices [25], while flexible glass as a photonic technology [Contribution 6] shows how ultrathin glass can provide bendability, durability, and optical functionality for flexible photonic platforms. These examples suggest that materials are judged not by their intrinsic properties alone but by how well they generate, detect, and deliver optical function in real operating environments.

## 3. Conclusions and Outlook

Overall, these contributions point to a coherent direction: optical and photonic materials are increasingly being designed as active, reconfigurable, and structurally engineered platforms rather than as fixed media. Their value is also judged by how well they perform in integrated devices and realistic operating environments. Two cross-cutting needs stand out.

First, control requires characterization that can keep pace with material and device complexity. Plasmonic imaging of single graphene and graphene oxide sheets [26], for example, provides a fast and non-destructive route for morphology mapping of graphene-based sheets. Related broadband terahertz spectroscopy of single-crystal diamond [27] further shows how spectroscopic measurements can resolve coupled carrier and polarization dynamics that narrower measurement windows may miss. In a different context, the reassessment of thermally enhanced upconversion luminescence in lanthanide-doped nanosized fluoride phosphors [Contribution 7] highlights how apparent optical enhancement can be influenced by multiple extrinsic factors, including moisture desorption, laser-induced local heating, and lattice thermal expansion. Together, these examples reinforce the importance of reliable measurement and careful separation of intrinsic material response from experimental artifacts.

Second, future progress will likely depend on connecting these themes more directly. Tunable materials can be combined with symmetry-based design, low-dimensional crystals can be integrated into working devices, and material platforms should be developed together with suitable characterization methods and application requirements. The articles collected here do not resolve all of these challenges, but they point to useful directions for designing optical and photonic materials that can operate more reliably in practical device environments.
